# Association of variants in *BAFF* (rs9514828 and rs1041569) and *BAFF-R* (rs61756766) genes with the risk of chronic lymphocytic leukemia

**DOI:** 10.1007/s13277-016-5182-z

**Published:** 2016-07-29

**Authors:** Monika Jasek, Agnieszka Bojarska-Junak, Marta Wagner, Maciej Sobczyński, Dariusz Wołowiec, Jacek Roliński, Lidia Karabon, Piotr Kuśnierczyk

**Affiliations:** 1 Laboratory of Immunogenetics and Tissue Immunology, Department of Clinical Immunology, Ludwik Hirszfeld Institute of Immunology and Experimental Therapy, Polish Academy of Sciences, ul. Weigla 12, 53-114 Wroclaw, Poland; 2 Chair and Department of Clinical Immunology, Medical University of Lublin, Chodzki 4a, 20-093 Lublin, Poland; 3 Department of Genomics, Faculty of Biotechnology, University of Wrocław, ul. Fryderyka Joliot-Curie 14a, 50-383 Wroclaw, Poland; 4 Department of Hematology, Neoplastic Diseases, and Bone Marrow Transplantation, Wroclaw Medical University, ul. Pasteura 1, 50-367 Wroclaw, Poland; 5 Department of Experimental Therapy, Institute of Immunology and Experimental Therapy, Polish Academy of Science, ul. Weigla 12, 53-114 Wroclaw, Poland

**Keywords:** CLL, BAFF, BAFF-R, Polymorphism

## Abstract

The B-cell activator factor (BAFF)/BAFF receptor (BAFF-R) axis seems to play an important role in the development and progression of chronic lymphocytic leukemia (CLL). Here, we investigated the association of eight single nucleotide polymorphisms (SNPs) in the *BAFF* (TNFSF13B) and *BAFF-R* (TNFRSF13C) genes with risk of sporadic CLL in a group of 439 CLL patients and 477 controls. We also examined the correlation between selected SNPs and CLL clinical parameters as well as BAFF plasma levels and intracellular BAFF expression. Our results point to a possible association between the rs9514828 (CT vs. CC + TT; OR = 0.74; CI 95 % = 0.57; 0.97; *p* = 0.022) and rs1041569 (AT vs. AA + TT; OR = 0.72; CI 95 % = 0.54; 0.95; *p* = 0.021) of *BAFF* gene and rs61756766 (CC vs. CT; OR = 2.03; CI 95 % = 1.03; 3.99; *p =* 0.03) of *BAFF-R* gene and CLL risk. Additionally, we observed that homozygotes rs1041569 AA and TT had a slightly higher risk (HR = 1.12) for the need of treatment in comparison to AT heterozygotes. In conclusion, our results indicate that SNPs in *BAFF* and *BAFF-R* genes may be considered as potential CLL risk factors.

**Electronic supplementary material:**

The online version of this article (doi:10.1007/s13277-016-5182-z) contains supplementary material, which is available to authorized users.

## Introduction

Chronic lymphocytic leukemia (CLL) is one of the most prevalent leukemias in Western countries [1] with an incidence of 4.1 per 100,000 persons per year [2]. The characteristic feature of this disease is the gradual accumulation of mature B cells presenting typical markers such as CD5, CD19, CD20, and CD23 in lymphoid tissues, bone marrow, and peripheral blood (PB) [1, 3]. The CLL cell accumulation is caused by the disruption of programmed cell death rather than acute proliferation [1, 3].

B-cell activator factor (BAFF) (other names: BLys, TNFSF13B) and some other members of the tumor necrosis factor (TNF) family proteins have been shown to be engaged in providing survival signals to B cells by affecting genes associated with apoptosis [3]. BAFF binds to the following receptors: B cell maturation antigen (BCMA; TNFRSF17), transmembrane activator and calcium-modulator and cyclophilin ligand interactor (TACI; TNFRSF13B), and BAFF receptor (BAFF-R; TNFRSF13C) [4] which is a specific receptor for BAFF [5] and plays a key role in its biology [6].

BAFF-R is mainly expressed on B cells [6], and its expression on various B cell lines strongly correlates with BAFF binding to these cell lines [5]. Moreover, BAFF/BAFF-R signaling plays a central role in the survival and growth of normal and neoplastic B lymphocytes [5]. The aberrant expression of BAFF and BAFF-R has been reported, among others, in non-Hodgkin lymphomas (NHLs) including CLL [3, 5]. The SNP array designed to screen, inter alia, genes from TNF and TNF receptor superfamilies, as well as NFκB and related transcription factors, delineated *BAFF* (*TNFSF13B*) and *BAFF-R* (*TNFRSF13C*) genes as associated with NHL risk. Moreover, analysis conducted by NHL subtype revealed *BAFF* (*TNFSF13B*) to be associated specifically with CLL/small lymphocytic lymphoma (SLL) [7].

On the basis of these data, the hypothesis that the BAFF/BAFF-R pathway may play a role in the development and pathogenesis of this disease has been put forward [5].

The following observations support this hypothesis. Aberrant serum soluble BAFF levels have been reported in NHL patients including CLL patients [5, 8–11]. Correlations between BAFF expression and some clinical prognosis markers have been reported [5], and, what is more, the use of BAFF serum levels together with CD38, ZAP70 expression, and immunoglobulin heavy chain variable (IGHV) mutational status has been presented as a promising marker in CLL [12].

Genetic evidence also supports the above-mentioned hypothesis. Polymorphism rs9514828 (−871 C>T) in the promoter region of the *BAFF* gene has been associated with high levels of serum BAFF and familial CLL [3] and NHL risk [13]. A new mutation in the *BAFF-R* gene rs61756766 (His159Tyr) was determined in tumor and germline tissues from a subset of NHL patients [5, 6]. Additionally, the results of our preliminary study pointed to a possible association between genetic variations of *BAFF* and *BAFF-R* genes and the risk of sporadic CLL [14].

Since the BAFF/BAFF-R axis seems to play an important role in the development and progression of CLL, we decided to extend our previous study to investigate the association between single nucleotide polymorphisms (SNPs) of the *BAFF* and *BAFF-R* genes and CLL risk. We also examined the correlation between these SNPs and CLL clinical parameters as well as BAFF plasma level and intracellular BAFF protein expression.

## Materials and methods

### Study population

Patient population (*N* = 439) was composed of two cohorts of CLL patients. The majority of the first group (193 CLL patients; 85 females and 108 males) had entered into our previous study [14]. These patients were diagnosed with CLL in the Department of Hematology, Neoplastic Disease, and Bone Marrow Transplantation of Medical University of Wroclaw. The second group consisted of 246 patients (107 females and 139 males) diagnosed in the Department of Hematooncology and Bone Marrow Transplantation of Medical University of Lublin. Table 1 contains characteristics of CLL patients. Diagnosis of CLL was based on criteria from the International Workshop on Chronic Lymphocytic Leukemia (IWCLL) [15]. The follow-up period of this group was from 2 to 171 months (means *±* SD = 58.01 *±* 33.99). During this period, 106 patient indications for cytostatic treatment according to the IWCLL [15] were established. The period from the enrolment into the study and treatment ranged from 0.5 to 123 months (mean *±* SD = 14.14 *±* 21.75).

**Table 1:** Characteristics of CLL patient group

Variables	Min	Q1	Median	S_n_	Q3	Max
Age at diagnosis	31	55.5	64	10	71	91
WBC	3.25	18.4	30.36	18.38	56.02	387.2
LIMF	2.12	12.48	22.24	14.99	46.4	232
HGB	7.04	12.1	13.4	1.3	14.4	18.1
PLT	17	133	178	50	214	626
B2M	0.56	2.135	2.735	0.94	3.828	12.79
LDH	1	39.25	72	40	107.8	163
Variables	Yes	No	Σ	% Yes	CI 95 %	
Woman	192	247	439	43.7	39	48.5
Therapy	106	140	246	43.1	36.8	49.5
ZAP70^a^	88	158	246	35.8	29.8	42.1
CD38^a^	81	165	246	32.9	27.1	39.2
CD5^a^	239	7	246	97.2	94.2	98.8
Rai	0	I	II	III	IV	Σ
N	135	111	100	26	67	439
%	30.8	25.3	22.8	5.9	15.3	100 %
cum. %	30.8	56.1	78.9	84.8	100 %	–

Not all clinical data were available for all of the patients

*Q1* 1st quartile, *Q3* 3rd quartile, *S*_*n*_ variability measure, *WBC* while blood cell (G/L), *LIMF* lymphocyte count (G/L), *HGB* hemoglobin (g/dl), *PLT* platelets (G/L), *LDH* lactate dehydrogenase (IU/L), *B2M* β2-microglobulin (mg/dl), *Rai* Rai stage

^a^Cutoff (20 %)

The control population was composed of 477 (296 subjects from our previous study and 181 additional subjects) randomly selected blood donors of Polish Caucasian origin (243 females and 234 males).

This study was approved by the Ethics Committee of the Medical University of Wroclaw and the Ethics Committee of the Medical University of Lublin. Written informed consent was obtained from all participants.

### Selection of single nucleotide polymorphisms

SNPs, as previously described [14], were selected based on available literature [6, 16] and in silico analysis [17, 18].

The following SNPs of the 5′-untranslated region (UTR) of *BAFF* (*TNFSF13B*; 13q33.3) were examined: rs9514827 T>C (−2841); rs3759467 T>C (−2704); rs1041569 A>T (−2701); and rs9514828 C>T (−871) [14, 16]. According to in silico analysis, all these SNPs are located in the potential transcription factors binding sites (TFBS) [14, 17, 18].

As previously [14], we investigated four SNPs of *BAFF-R* (*TNFRSF13C*; 22q13.2): rs5996088 G>A (5′-near gene; potential TFB site); rs61756766 C>T (His159Tyr) [6] (Online Resource—Supplementary Fig. 1); rs7290134 T>C (3′-UTR; the potential miRNA binding site), and rs6002551 C>T (described by Wang et al. as a polymorphic variant of *BAFF-R* associated with NHL (7).

### DNA isolation and genotyping

Genomic DNA was isolated from whole blood using Invisorb Blood Midi Kit (Stratec Molecular GmbH, Berlin, Germany) according to the manufacturer’s protocol. The following SNPs were genotyped by restriction fragment length polymorphism (RFLP): *BAFF* (rs1041569; rs9514827) [14, 16] and *BAFF-R* (rs61756766; rs6002551) [6, 14]. Primer sequences, annealing temperatures, and restriction enzymes are listed in Online Resource (Supplementary Table 1). Polymerase chain reactions (PCRs) were run on T100™ Thermal Cycler (Bio-Rad, Hercules, CA, USA). Allelic discrimination method with application of TaqMan SNP Genotyping Assays (Thermo Fisher Scientific, Waltham, MA, USA) was used to determine the following SNPs: *BAFF* (rs3759467, assay ID C_27497010_10) and *BAFF-R* (rs7290134, assay ID C_2189968_1_; rs5996088, assay ID C_30413471_10) [14]. The reactions were run on Applied Biosystems 7300 Real-Time PCR System (Thermo Fisher Scientific, Waltham, MA, USA (Online Resource—Supplementary Table 2 contains a detailed list of assays used in this study). The *BAFF* rs9514828 was examined by both RFLP [16] (*AciI*, catalog no. R0551, New England Biolabs® Inc., Ipswich, MA, USA) and TaqMan methods (assay ID C_29641742_10) or by double genotyping with a TaqMan probe. Accuracy of genotyping methods for all SNPs was verified by direct sequencing of a few samples representing homozygotes of two types and heterozygotes for each investigated SNP. These samples were used as reference samples in the following genotyping experiments.

### Intracellular analysis of BAFF

Peripheral blood samples from CLL patients were stained for intracellular BAFF expression as previously described [8]. Briefly, analysis of intracellular BAFF expression by CD19+ cells was performed on fresh PB samples. Monoclonal antibodies (MoAbs) used for analyses included anti-CD19 PE (Clone HIB 19, IgG_1_, BD Pharmingen, San Diego, CA, USA) and fluorescein isothiocyanate (FITC)-conjugated anti-BAFF (Clone 137314, IgG_1_, R&D Systems, Minneapolis, MN USA). The samples were analyzed by flow cytometry directly after preparation. For data acquisition and analysis, a FACSCalibur instrument with CellQuest software (Becton Dickinson, Franklin Lakes, NJ, USA) was used. The percentage of positive cells was measured from a cutoff set using an isotype-matched nonspecific control antibody.

### Analysis of ZAP-70 expression in CLL cells

CLL cells were stained for ZAP-70 protein expression as previously described [8]. Briefly, ZAP-70-positive cells were evaluated in the PB via analysis of the surface expression of CD19 and CD5 antigens, as well as intracellular expression of ZAP-70 by flow cytometry. The percentage of CD19+CD5+ZAP-70+ cells was determined. MoAbs used for analyses included anti-ZAP-70 PE (Clone 1E7.2, IgG1, BD Biosciences, Franklin Lakes, NJ, USA) and anti-CD19 FITC (Clone HIB19, IgG1) and anti-CD5 PE-Cy5 (Clone UCHT2, IgG1) (BD Pharmingen, San Diego, CA, USA). The cutoff point for ZAP-70 positivity in leukemic cells was ≥20 %.

### Detection of CD38 expression

As previously described [8], flow cytometry analysis of CD38 was performed on fresh PB samples stained with anti-CD38 FITC (Clone HIT2, IgG_1_), anti-CD5 PE-Cy5, and anti-CD19 PE (BD Pharmingen, San Diego, CA, USA). A standard, whole-blood assay with erythrocyte cell lysis was used for preparing the PB specimens. The samples were analyzed by flow cytometry directly after preparation. CLL cells were considered CD38-positive when ≥20 % was expressed in the membrane antigen.

### Plasma BAFF immunoassay

As previously described [8], a commercial enzyme-linked immunosorbent assay (ELISA) kit, Quantikine Human BAFF/BLyS Immunoassay (R&D Systems, Inc. Minneapolis, MN, USA), was used for quantitative determination of human BAFF in plasma samples. We followed the protocol recommended by the manufacturer. The ELISA Reader Elx800 (BioTek Instruments, Winooski, VT, USA) was used [8].

### Statistical analysis

As in our preliminary study [14], chi-square test, *χ* ^2^_df_, was used to test the null hypothesis that cases and controls have the same distribution of genotype counts. To control type I error in the case of many tests for differences between SNP genotypes of cases and controls, a global (omnibus) chi-square test was performed, first to test the hypothesis zero, H0, which states that there were no differences between cases and controls in any SNP, opposite to the alternative, H1, which states that genotype frequencies in cases and controls were different at least in one SNP. Due to correlation between SNP distributions (linkage disequilibrium present), the distribution of global chi-square statistic was estimated numerically. In the case of small numbers, test statistics distribution was estimated numerically. *Odds ratio* (OR) was computed as the measure of effect size. Median was used as the location parameter. In the case of the median, the *S*_*n*_statistic was computed as the measure of variability: *S*_*n*_ = *med*{*med*|*x*_*i*_ − *x*_*j*_|; *j* = 1 ... *n*} [19]. *S*_*n*_ is the typical distance between two randomly selected individuals and is used as the measure of variability instead of standard deviations when median is used instead of arithmetic mean. Additionally, the 1st and 3rd quartiles and minimal and maximal observations were reported. A linear model was used to test relations between SNP haplotypes and time to treatment (TTT) and requirement for introducing treatment. The likelihood ratio statistic, LRS ∼ *χ* ^2^, was used to test regression coefficients. The difference between two medians of plasma BAFF level was tested based on the bootstrapped studentized T statistic. Deviation from the Hardy-Weinberg equilibrium (HWE) was estimated with the chi-square test and measured as $f=\frac{p_{CC}-{p}_C^2}{p_C\left(1-{p}_C\right)}$, where *p*_*C*_ and *p*_*CC*_ are allele *C* and genotype *CC* frequencies while *f* < 0 and *f* > 0 correspond to deficiency and excess of homozygotes, respectively, and *f* = 0 in the case of HWE. Haplotype frequencies (HFs) among SNPs were estimated with the *maximum likelihood* function [20]. Differences in genotype distributions between two groups of cases and between combined group of cases and controls were tested with the χ^2^ statistic, and the distribution of χ^2^ was estimated numerically. Analysis was performed using GNU Octave software version 3.8.2.

## Results

The case-control study was carried out on groups of patients and controls described in the “Study population” section. There was no evidence that two groups of patients were different in terms of genotype distribution for any of the examined SNPs (*χ* ^2^_df≈39_ = 43.97, *p* = 0.2673), so it allowed us to combine patients groups (193 + 246) in order to increase the statistical power of the present case-control study. The global (omnibus) test for homogeneity (*χ* ^2^ = 55 on approximately 35 degrees of freedom, ratio *χ* ^2^/df = 1.57) showed that the CLL group differed from the healthy control (HC) group in at least one SNP (*χ* ^2^_df=35_ = 55; *p* = 0.026).

### Hardy-Weinberg equilibrium

Seven out of eight SNPs investigated in this study were in the Hardy-Weinberg equilibrium in HC (Table 2 and Online Resource—Supplementary Table 3). The genotype distribution for rs9514828 differed from that expected under HWE in HC (*p* = 0.015; *f* = −0.116; CI 95 % = −0.21, −0.03). We did not exclude this variant from the analysis for several reasons. This particular SNP is functional and was the only one out of 20 SNPs examined (this manuscript presents only results for BAFF and BAFF-R) in our HC group for which deviation from HWE (DHW) was observed. It is well established that the most common sources of deviation from HWE are genotyping errors or population stratification [21]. Genotyping errors were excluded by double genotyping and by the fact that the patient group was in HWE. It is also hardly likely that our study population was stratified resulting from the admixture of other ethnic groups since both of the investigated groups are of Polish origin and they did not differ in terms of allele frequency. The C and T alleles frequencies were as follows: HC patients, C—56.9 %, T—43.1 %; and CLL patients, C—56.8 %, T—43.2 %.

**Table 2:** Genotype distribution of the *BAFF* (*TNFSF13B*) rs1041569 and rs9514828 polymorphisms and *BAFF-R* (*TNFRSF13C*) rs61756766 polymorphism in CLL patients and controls

*BAFF TNFSF13B* polymorphisms		Patients (*N* = 439)			Controls (*N* = 477)			OR	CI 95 %		Patients vs. controls
		*N*	%	HWE	*N*	%	HWE				
rs1041569 -2701	AA	303	69.00	*p* = 0.015	309	64.80	*p* = 0.098	1^a^			*χ* ^2^_df=1_ = 5.29 *p* = 0.021
	AT	114	26.00	*f* = 0.12	157	32.90	*f* = −0.08	0.74	0.56	0.99	
	TT	22	5.00	CI 95 % = 0.013, 0.22	11	2.30	CI 95 % = −0.15, 0.003	2.00	0.96	4.13	
rs9514828 -871	CC	146	33.30	*p* = 0.436	141	29.60	*p* = 0.015	1^a^			*χ* ^2^_df=1_ = 5.23 *p* = 0.022
	CT	207	47.20	*f* = 0.039	261	54.70	*f* = −0.116	0.77	0.57	1.03	
	TT	86	19.60	CI 95 % = −0.06, 0.13	75	15.70	CI 95 % = −0.21, −0.03	1.11	0.75	1.63	
BAFF-R *TNFRSF13C* polymorphisms		Patients (*N* = 439)			Controls (*N* = 477)			OR	CI 95 %		Patients vs. controls
		N	%	HWE	N	%	HWE				
rs61756766 His159Tyr	CC	415	94.50	*p* = 0.811	464	97.30	*p* = 0.898	1^*^			*χ* ^2^_df=1_ = 4.43 *p* = 0.035
	CT	24	5.50	*f* = −0.03	13	2.70	*f* = −0.014	2.03	1.03	3.99	
	TT	0	0.00	CI 95 % = −0.04, −0.02	0	0.00	CI 95 % = −0.021, −0.007	1.12	0.02	56.47	

*OR* odds ratio, *CI* confidence intervals, *HWE* test for Hardy-Weinberg equilibrium, *f* departure from HWE

^a^The reference group

From the statistical point of view, it is not surprising that one out of 20 SNPs tested at *α* = 0.05 was not in HWE, and as a matter of fact it is expected, even if all 20 examined SNPs in the general population were in HWE. Let *s* be the number of examined SNPs which are not in HWE in the sample at *α* = 0.05 and let *r* be the number of examined SNPs which are in HWE in the general population. We then get *P*(*s* ≥ 1| *r* = 20, *α* = 0.05) = 0.6415.

### BAFF and BAFF-R polymorphisms and risk of CLL

In our preliminary study, we observed a difference in genotype distribution between the CLL patients and the controls for the rs9514828 *BAFF* variant (*χ* ^2^_df=1_ = 3.946; *p =* 0.047) [14]. Here, in larger groups, we confirmed this difference (*χ* ^2^_df=1_ = 5.23; *p =* 0.022) (Table 2). The risk of CLL for rs9514828 CT heterozygotes was lower than that for homozygotes CC (OR = 0.77; CI 95 % = 0.57, 1.03), while for rs9514828 TT homozygotes this risk was almost the same as for rs9514828 CC homozygotes (OR = 1.11 CI 95 % = 0.75, 1.63). Therefore, according to the parsimony rule, we assumed the overdominant model. The conducted analysis confirmed our assumption. In this model, heterozygotes had a protective effect (CT vs. CC + TT; *χ* ^2^_df=1_ = 5.229; OR = 0.74; CI 95 % = 0.57, 0.97; *p* = 0.022).

In addition, we observed a difference in genotype distribution between the CLL patients and the controls for rs1041569 (*χ* ^2^_df=1_ = 5.29; *p =* 0.02). Similarly, the genotype rs1041569 AT was protective when compared to rs1041569 AA (OR = 0.74; CI 95 % = 0.56, 0.99) (Table 2). The overdominant model applied in this case showed protective effect of heterozygote AT (AT vs. AA + TT; *χ* ^2^_df=1_ = 5.886; OR = 0.72; CI 95 % = 0.54, 0.95; *p* = 0.021).

In our preliminary study [14], we observed a higher risk of CLL for rs61756766 CT heterozygotes, a very rare variant of the *BAFF-R* gene (OR = 1.79; CI 95 % = 0.73, 4.39), but the power of that study was low. Here, on larger groups, we noted a significant difference in genotype distribution between patients and controls (*χ* ^2^_df=1_ = 4.43; *p =* 0.03) (Table 2). Moreover, we found the association between the rs61756766 CT genotype and the risk of CLL (OR = 2.03; CI 95 % = 1.03, 3.99). As previously [14], we did not determine any TT homozygote in patients or in controls.

Supplementary Table 3 (Online Resource) shows the distribution of the *BAFF* rs9514827 and rs3759467 and *BAFF-R* rs6002551, rs5996088, and rs7290134 gene polymorphisms investigated in this study for which distribution of genotypes was similar in patients and controls.

Additionally, we compared the distribution of *BAFF* and *BAFF-R* haplotypes between patients and controls. We noted a significant difference in haplotype distribution between HC and CLL subjects for *BAFF-R* (*χ* ^2^_df=6_ = 17.4; *p =* 0.008) (Online Resource; Supplementary Table 4). There was no *gene × gene* interaction between *BAFF* and *BAFF-R* associated with the risk of CLL.

### Plasma BAFF, intracellular expression of BAFF, and polymorphisms of BAFF

We investigated the possible association between all *BAFF* SNPs analyzed in this study as well as the (1) plasma BAFF level and the (2) intracellular expression of BAFF protein in PB CD19^+^ cells. We failed to find any of the *BAFF* SNPs examined in this study to be associated with plasma BAFF level (*F*_df=8155_ = 1.154, *p* = 0.331) (Online Resource; Supplementary Table 5) or to be associated with intracellular expression of BAFF in PB CD19^+^ cells (*F*_df=8112_ = 1.166, *p* = 0.326) (Online Resource; Supplementary Table 6).

### BAFF and BAFF-R polymorphisms and clinical parameters

Since CD38 and ZAP70 are important prognostic markers of CLL, we divided patients into the following groups (using a 20 % cutoff value for CD38 and ZAP70): CD38^+^ and CD38^−^, and ZAP70^+^ and ZAP70^−^. Next, we compared the genotype distribution of all investigated SNPs of *BAFF* and *BAFF-R* between CD38^+^ and CD38^−^ and ZAP70^+^ and ZAP70^−^ CLL patients, but we did not find any significant differences. Also, none of the examined SNPs was associated with Rai stage.

Next, we analyzed correlations between the appearance of cytostatic treatment indications and time to treatment during the follow-up period of 2 to 171 months as well as the (1) haplotypes of BAFF and (2) rs1041569 and (3) rs9514828 of *BAFF*. There was no association between the requirement of the treatment and haplotypes (*χ* ^2^_df=8_ = 4.38; *p =* 0.82) (Online Resource; Supplementary Table 7) as well as between treatment requirement and individual SNPs (rs1041569 *χ* ^2^_df=1_ = 0.77; *p =* 0.68; rs9514828 *χ* ^2^_df=1_ = 0.51; *p =* 0.48). Similarly, we did not observe a relationship between TTT and haplotypes of *BAFF* (Online Resource; Supplementary Table 8) (*χ* ^2^_df=6_ = 5.97; *p =* 0.43).

**Fig. 1:**
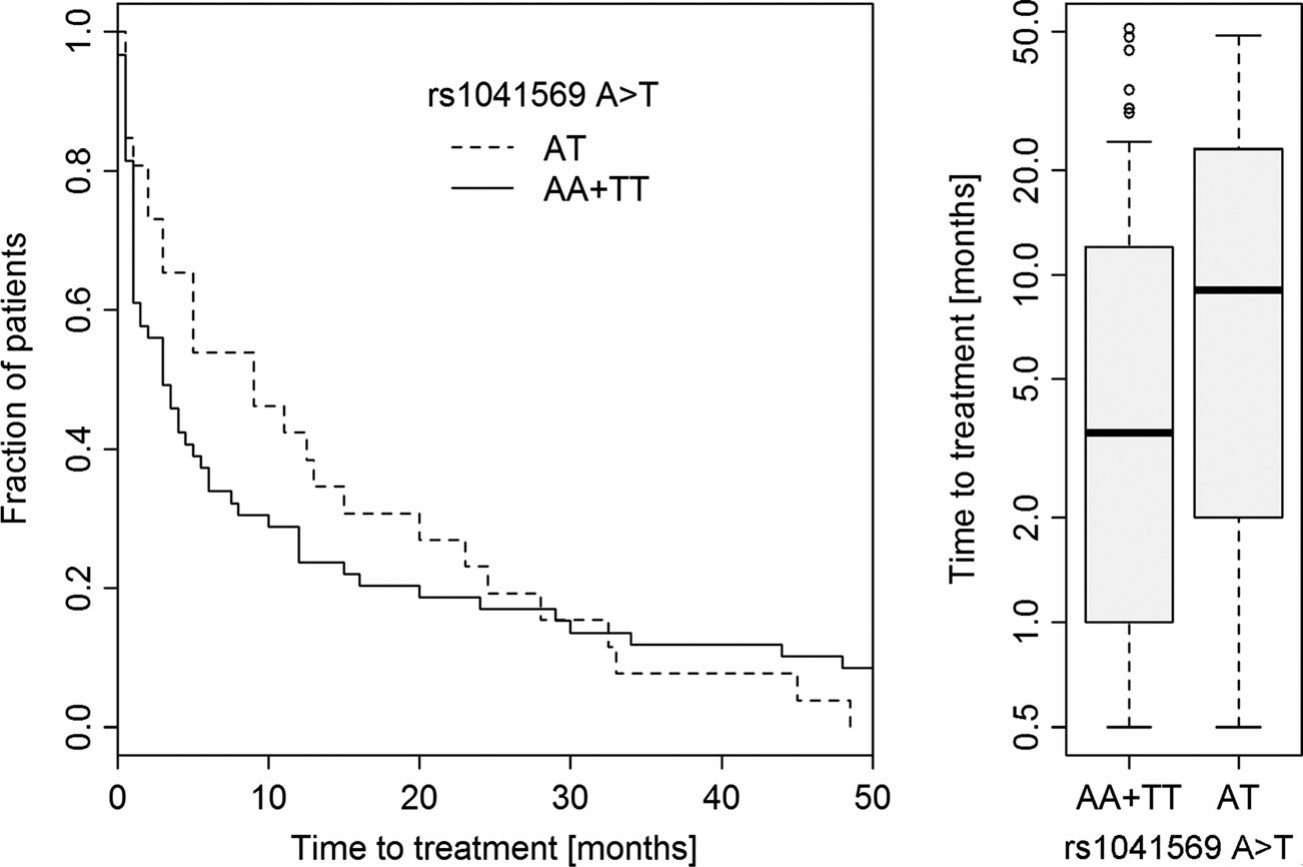
Kaplan-Meier estimates of the time to treatment of CLL patients in relation to rs1041569 genotypes

Since we noticed that heterozygotes rs9514828 CT and rs1041569 AT had lower risk of CLL, in the next stage we analyzed TTT in relation to heterozygosity at rs1041569 and rs9514828 *loci* of the *BAFF* gene (Table 3). We noted that the average TTT for the rs1041569 AT heterozygotes was longer than in the group of AA and TT homozygotes. Homozygotes had 1.12 (HR = 1.12) times higher risk for the need of treatment (*p* = 0.316; CI 95 % = 0.7, 1.79). This result is encouraging because in practice it means that the TTT for patients carrying the AT genotype is more than two times longer than for patients carrying the AA or TT genotype (Fig. 1.) We also checked whether the heterozygotes at rs9514828 and rs1041569 would have longer overall survival (OS) in comparison to homozygotes, but we did not observe such a relation (*p* = 0.851 and *p* = 0.957, respectively).

**Table 3:** The time to treatment in relation to heterozygosity in rs1041569 and rs9514828 *loci* of *BAFF* gene

SNP	Genotyp	N	Min	Q1	Median	S_n_	Q3	Max
rs1041569	AA + TT	59	0	1	3	3	12	123
	AT	26	0.5	2.3	9	8.5	22.3	48.5
rs9514828	CC + TT	59	0.5	1	4	3.8	11.5	48.5
	CT	26	0	1	4	4.8	21.3	123

*N* number of patients, *Min* minimum, *Q1* 1st quartile, *Q3* 3rd quartile, *S*_*n*_ variability measure, *Max* maximum

## Discussion

In our previous preliminary study, we investigated 20 SNPs of the BAFF/APRIL system and their association with CLL risk and some clinical parameters [14]. Here, we examined the association of *BAFF* and *BAFF-R* polymorphisms and CLL risk on a much larger group of patients (*N* = 439) and controls (*N* = 477) to confirm our previous findings [14].

The *BAFF* gene rs9514828 is located within the binding site of the myeloid zinc finger protein 1 (MZF1) transcription factor (TF) [9], and its functionality has been shown in luciferase assays, where the rs9514828 T allele was associated with higher luciferase activity [9, 22]. In contrast, however, Almeida and Petzl-Erler [23] who investigated BAFF expression by unstimulated cells (CD8+ T cells, monocytes, and NK cells) isolated from healthy individuals at protein and mRNA levels found that the rs9514828 CC genotype was associated with significantly higher BAFF expression.

The association of rs9514828 with different diseases such as primary Sjögren’s syndrome (pSS) [24], familial CLL [9], idiopathic thrombocytopenic purpura [25], hepatitis C virus-associated mixed cryoglobulinemia [26], pemphigus foliaceus [27], and T-cell lymphomas [22] has been reported. Of note, the protective or risk effect for C and T alleles has been shown for different diseases [9, 14, 22–25, 27].

In our previous study, we observed the difference in rs9514828 genotype distribution between CLL patients and HC (*p =* 0.047) [14].

Due to the above-mentioned contradictory data regarding the association of the rs9514828 variant with different diseases and the association of the T and C alleles with aberrant *BAFF* expression and our own data, we were prompted to verify these observations on larger groups of healthy and CLL subjects.

We confirmed our previous finding showing the difference in genotype distribution between CLL patients and controls for rs9514828. Based on obtained OR values, we assumed the overdominant model in which heterozygotes had protective effect.

As was mentioned earlier, rs9514828 is located within the MZF1-binding site [9]. We ran an in silico analysis using ConSite software [28] and found that the binding of MZF1 is predicted to be less strong for the C allele (score 8.510) than for the T allele (score 9.085). MZF1 has been shown to be involved in hematopoietic malignancies. MZF1 is a promoter/enhancer binding-type transcription factor and has been shown to act as a *trans* activator as well as a *trans* repressor, and it has been suggested that its oncogenic activity may be related to combined values of increased and decreased expression of target genes [29]. Moreover, ConSite software predicted the presence of a potential binding site for Sp1 transcription factor but only for the C allele (score 6.115). One of the mechanisms allowing neoplastic cells to survive and develop is the ability to overcome the intrinsic and extrinsic signals which in normal conditions lead to apoptosis. Sp1 transcription factor has been shown to be involved in regulation of numerous pro- and anti-apoptotic proteins such as BCL-2 or MCL1. It has also been reported that Sp1 regulation may act in two opposite directions. It may promote neoplastic cell resistance to apoptosis but may also promote these cells’ sensitivity to induction of apoptosis [30].

Taking into consideration the results of the in silico analysis, one may speculate that rs9514828 polymorphism may influence the regulation of the *BAFF* gene by affecting the binding sites of the above-mentioned transcription factors. The normal and neoplastic cells may differ in terms of expression level of these transcription factors which may influence the BAFF regulation.

Since both C and T alleles have been shown to have a different effect of BAFF expression in neoplastic cells, cell lines, and cells isolated from healthy donors and have been found to be predisposing or protecting in different diseases, it cannot be excluded that having both alleles may be advantageous by allowing cells to adjust to normal and/or abnormal conditions. Further research is required to test our hypothesis. First of all, it has to be investigated if the predicted in silico binding sites for transcription factors are functional. Subsequently, it has to be checked if and how these TFs regulate BAFF. Also, the expression level of MZF1 and Sp1 factors should be compared between CLL and normal cells.

According to our best knowledge, apart from our studies (present and preliminary [14]) in the literature there is only one other study investigating the association of *BAFF* gene polymorphism rs9514828 with sporadic CLL [10]. Novak et al. [10] did not find this variant to be associated with the risk of NHL or CLL. However, that study involved only 123 CLL/SLL cases [10]. Due to the discrepancy between this and our study, the association of rs9514828 with the risk of CLL remains to be examined in additional, independent studies. It needs to be clarified, whether this SNP is a true risk variant of CLL and if it is specific only for familial CLL [9] or both sporadic and familial CLL.

Apart from the association between rs9514828 and CLL, we also observed a significant difference in genotype distribution between CLL patients and controls for rs1041569*.* In the overdominant model, the AT heterozygotes showed a protective effect. Faustova et al. [31] reported a significant association of the rs1041569 T allele with myositis [31]. Nezos et al. [24] investigated rs1041569 polymorphism in primary Sjögren’s syndrome, but this group excluded this SNP from most of the conducted analysis since it was not in HWE in the healthy control group [24]. Recently, Lin et al. [32] have investigated rs1041569 in autoimmune thyroid diseases (Grave’s disease and Hashimoto’s thyroiditis). They did not find an association between this SNP and the examined diseases, but OR and CI 95 % reported by this group for rs1041569 AT heterozygotes (OR = 0.79; CI 95 % 0.57, 1.10) [32] were similar to the figure observed by us (OR = 0.74; CI 95 % = 0.57, 0.97). It is difficult to draw any definite conclusion regarding the ORs for TT homozygotes based on our and Lin et al.’s [32] studies due to the low frequency of this genotype observed in both populations as well as the CI 95 % values (our study, 2.3 %; CI 95 % = 0.96, 4.13; Lin et al.’s study [32], 1.1 %; CI 95 % = 0.14, 2.93).

The in silico analysis via application of ConSite [28] and SNPinfo [17] showed that this variant is located within the E47 (Thing1/E47) TFBS. The calculated matrix similarity for E47 (Thing1/E47) at position rs1041569 suggests better binding of this TF to the A allele (ConSite scores for A allele 9.982 and 8.897 for T allele; SNPinfo: A allele core similarity 1 and matrix similarity 0.928; T allele core similarity 1 and matrix similarity 0.911).

E12 and E47 are products of the alternative splicing of a single *TCF3* (*E2A*) gene. They belong to the class I helix-loop helix (bHLH) proteins and are broadly expressed, multifunctional TFs playing a role in many developmental processes [33, 34]. These two proteins can act both as tumor suppressors and as tumor promoters [35]. E2A (E47) TF plays a key role in B-cell development, maturation, and function and regulates proliferation and survival of these cells [36].

It has been shown that E2A (E47) is overexpressed in CLL cells. Elimination of this TF caused increased apoptosis [36]. Additionally, ConSite prediction showed that the T allele caused the disappearance of the GATA-2 binding site (score 4.174 for A allele) and introduced the possible binding site for PU.1 (SPI-1 score 4.776). However, due to limited information available about the function of this SNP, it is difficult to propose the mechanism which will explain the observed protective effect for AT heterozygotes. As was mentioned above, the rs1041569 TT homozygotes are rare, and study on larger sample size of CLL patients and healthy controls is needed to verify the effect associated with this genotype and the risk of CLL. Moreover, our study is the first (according to our best knowledge) to report an association between rs1041569 and the risk of CLL, and as was mentioned above, further case-control studies on large groups are necessary.

The different softwares designed to predict potential TFBS provide results which have to be evaluated in functional studies. Therefore, to resolve all arisen discrepancies, further in silico, genetic and functional studies are needed.

As described above, we observed that heterozygosity at rs9514828 and rs1041569 *loci* of the *BAFF* gene may protect from CLL development. Therefore, we checked if rs9514828 CT and rs1041569 AT heterozygotes would have a better prognosis in terms of overall survival, time to treatment, or the requirement for introducing treatment. Neither rs9514828 CT nor rs1041569 AT heterozygotes had longer overall survival in comparison to both types of homozygotes. There was also no association between the requirement for treatment and heterozygosity at rs9514828 or at rs1041569. No association was found between time to treatment and heterozygosity at rs1041569. However, we observed that the average time to beginning of treatment was much longer for rs1041569 AT heterozygotes in relation to homozygotes AA and TT. This result is in agreement with the lower risk of CLL for rs1041569 AT heterozygotes, observed here. We are not able to provide an explanation of this observation at this moment, but the influence from interactions between TFs and administered drugs cannot be excluded.

It has to be mentioned that we possessed a limited number of samples with TTT data available. It will be interesting to test our hypothesis of a protective role of heterozygosity at rs9514828 and rs1041569 variants of the *BAFF* gene on a larger group of CLL patients and HC.

As all *BAFF* SNPs analyzed in this study are located in potential TFBS and the relationship between rs9514828 polymorphism and expression level of BAFF was shown in luciferase assays [9, 22] and with serum BAFF levels (sBAFF) [9], we also addressed these issues in our research. We did not find any of the SNPs of the *BAFF* promoter investigated in the present study to be associated with plasma BAFF levels or with intracellular expression of BAFF protein in PB CD19^+^ cells. Similarly, Ansell et al. [37] did not find any significant association between rs9514828 and sBAFF levels in follicular grade 1 non-Hodgkin lymphoma [37]. The same result was obtained for pSS [16]. We examined the same SNPs of the *BAFF* promoter region (rs9514827, rs3759467, rs1041569, rs9514828) as had been previously studied by Eilertsen [38] and Fabris [39] and colleagues, who had investigated these SNPs in the systemic lupus erythematosus and rheumatoid arthritis, respectively. These authors also failed to find a relationship between serum BAFF levels and investigated promoter variants of the *BAFF* gene [38, 39]. Previously, Novak et al. [9] found that sBAFF levels were higher in patients with familial CLL than with sporadic CLL and suggested the correlation between elevated levels of serum BAFF and the presence of a T at rs9514828 in the *BAFF* promoter [9]. Since our cohort probably did not contain familial cases, we did not observe such a correlation in our study.

In silico analysis as well as previously mentioned results showing the association of rs9514828 with aberrant expression of *BAFF* gene suggested that we and other authors should observe a correlation between at least this variant and the level of *BAFF*. Due to the data available, we investigated only the correlation between promoter variants and *BAFF* protein levels. Any additional studies have to be designed to more deeply examine the role of SNPs investigated here in the regulation of the *BAFF* gene.

BAFF-R is the main receptor for BAFF, and the BAFF/BAFF-R pathway is crucial for the survival and growth of CLL cells [5]. Hildebrand et al. [6] described an association between the *BAFF-R* rs61756766 and the risk of non-Hodgkin lymphoma such as the diffuse large B cell lymphoma, follicular lymphoma, lymphoplasmacytic lymphoma, and mucosa-associated lymphoid tissue, but not with CLL. In the same publication, they also reported a correlation between this variant of the *BAFF-R* gene and the increased recruitment of TRAF 2, 3, and 6 and showed that signaling through this variant of *BAFF-R* resulted in increased NF-κB1 and NF-κB2 activity [6]. In our study, we found that rs61756766 CT heterozygotes had two times higher risk of CLL than CC homozygotes. Of note, we genotyped altogether 916 subjects (patients and controls) and did not find any rs61756766 TT homozygote. The frequency of the T allele was 2.7 % in patients and 1.4 % in HC. The absence of TT homozygotes was expected, since the probability that there will be no TT homozygotes within 439 cases and 477 controls given the above-mentioned T allele frequencies is *p* = 0.6592. Due to such low frequency of patients carrying the CT genotype for whom complete clinical data were available, we were not able to investigate potential relations between the presence of this allele and clinical features. Apart from rs61756766, we genotyped three additional SNPs of the *BAFF-R* gene: rs6002551, rs5996088, and rs7290134 [14]. We did not find any of these SNPs to be associated with the risk of CLL, but we observed a significant difference in haplotype distribution (formed by four SNPs of *BAFF-R*) between CLL and HC patients, which confirms that genetic predisposition to CLL may be associated with the *BAFF-R* gene.

In conclusion, in this study we investigated genetic variations in *BAFF* and *BAFF-R* in CLL including the assessment of haplotypes, *gene × gene* interaction, and correlation with clinical features. Our case-control study indicates a possible association of the most widely studied rs9514828 SNP of the *BAFF* gene as well as, described here for the first time, the possible association of rs1041569 of the *BAFF* gene with the risk of CLL. Moreover, this is the first study which showed the association between the *BAFF-R* gene rs61756766 and CLL risk. Taking into consideration the fact that genetic predisposition to CLL is still not well established, our results may help to further investigate this issue and may ultimately help to establish which of the genetic variations reported in the literature are the true CLL risk factors.

## Electronic supplementary material

ESM 1 (DOCX 102 kb)
