# Supplementary material for: Association of variants in BAFF (rs9514828 and rs1041569) and BAFF-R (rs61756766) genes with the risk of chronic lymphocytic leukemia
Source: Tumour Biol. 2016 Jul 29;37(10):13617–26. doi: 10.1007/s13277-016-5182-z (PMC5097080; doi:10.1007/s13277-016-5182-z)
Supplement: Supplementary file 1 — (DOCX 102 kb) [file 13277_2016_5182_MOESM1_ESM.docx]

**Association of variants in *BAFF* (-871 C>T; – 2701 A>T)** **and *BAFF-R* (His159Tyr) genes with the risk of chronic lymphocytic leukaemia**

**Journal name: *Tumor Biology***

Monika Jasek^a^, Agnieszka Bojarska-Junak^b^, Marta Wagner^a^, Maciej Sobczyński^c^, Dariusz Wołowiec^d^, Jacek Roliński^b^, Lidia Karabon^e^ and Piotr Kuśnierczyk^a^

^a^ Laboratory of Immunogenetics and Tissue Immunology, Department of Clinical Immunology, LudwikHirszfeld Institute of Immunology and Experimental Therapy, Polish Academy of Sciences, ul. Weigla 12, 53-114 Wroclaw, Poland

^b^ Chair and Department of Clinical Immunology, Medical University of Lublin, Chodzki 4a, 20-093 Lublin, Poland

^c^ Department of Genomics, Faculty of Biotechnology, University of Wrocław, ul. Fryderyka Joliot-Curie 14a, 50-383 Wroclaw, Poland

^d^ Department of Hematology, Neoplastic Diseases & Bone Marrow Transplantation, WroclawMedicalUniversity, ul. Pasteura 1, 50-367 Wroclaw, Poland

^e^ Department of Experimental Therapy, Institute of Immunology and Experimental Therapy, Polish Academy of Science, ul. Weigla 12, 53-114 Wroclaw, Poland

**Corresponding author:** Monika Jasek PhD; Laboratory of Immunogenetics and Tissue Immunology, Department of Clinical Immunology, Ludwik Hirszfeld Institute of Immunology and Experimental Therapy, Polish Academy of Sciences, ul. Weigla 12, 53-114 Wroclaw, Poland, Tel. +48 71 3709976; Fax +48-71-337 2171

E-mail address: [jasek@iitd.pan.wroc.pl](mailto:jasek@iitd.pan.wroc.pl)

Supplementary Table 1. Primer sequences, annealing temperatures and restriction enzymes used for PCR-RFLP genotyping.

| **SNP** | **Forward primer (5’-3’)** | **Reverse primer (5’-3’)** | **T_a_** | **Restriction enzyme** |
| --- | --- | --- | --- | --- |
| **rs9514827**^a^ | ATTCCCTGTCTTCAGAATTTTCTCT | CCTATAACTCCCACAATAAGGTGAC | 56˚C | *AciI* **^a^** |
| **rs1041569**^a^ | ATTCCCTGTCTTCAGAATTTTCTCT | CCTATAACTCCCACAATAAGGTGAC | 56˚C | *DpnII* **^a^** |
| **rs9514828** ^a^ | TTGTACACCGACCTGTTAGGC | TGGAAGTAAGTCCACTGGGAAT | 56˚C | *AciI* **^a^** |
| **rs61756766** | TCATTCTGTCTCCGGGAATC | GCTCCCTGCTATTGTTGCTC | 56˚C | *MlsI* (*MscI* ^b^) |
| **rs6002551** | CAGCAGGAGTTGATGTGCTC | GGTTCTCCCTGCATTTCATC | 62˚C | *TaqI* |

T_a_– annealing temperatures

^a^ Primers and enzymes were taken from Nossent et al. [16]

^b^According to Hildebrand et al. [6]

*AciI (Cat. # R0551); DpnII (Cat. # R0543)-* New England BioLabs® Inc., Ipswich, MA, USA;

*MlsI (MscI) (Cat.#* ER1211)*, TaqI* (*Cat.#* ER0671) - Thermo Fisher Scientific, Waltham, MA, USA

Supplementary Table 2. TaqMan SNP Genotyping Assays used for genotyping with applying the allelic discrimination method.

| **SNP** | **ID of TaqMan SNP Genotyping Assays** |
| --- | --- |
|  |  |
| **rs3759467** | C_27497010_10 |
| **rs9514828** | C_29641742_10 |
| **rs5996088** | C_30413471_10 |
| **rs7290134** | C_2189968_1_ |

Assays were purchased from Thermo Fisher Scientific, Waltham, MA, USA

Supplementary Table 3. Genotype distribution of the *BAFF* (*TNFSF13B*) and the *BAFF-R* (*TNFRSF13C*) polymorphisms in CLL patients and controls.

| ***BAFF***  ***TNFSF13B***  **polymorphisms** | | **Patients (N=439)** | |  | **Controls (N=477)** | |  | **OR** | **CI95%** | | **Patients vs. Controls** |  |
| --- | --- | --- | --- | --- | --- | --- | --- | --- | --- | --- | --- | --- |
|  |  | **N** | **%** | **HWE** | **N** | **%** | **HWE** |  |  |  |  |  |
| **rs9514827** | TT | 229 | 52.20 | p=0.549 | 256 | 53.70 | p=0.728 | 1^*^ |  |  | χ^2^_df=2_ =0.54  p=0.763 |  |
|  | TC | 180 | 41.00 | *f*=-0.032 | 185 | 38.80 | *f*=0.015 | 1.09 | 0.83 | 1.43 |  |  |
|  | CC | 30 | 6.80 | CI95%=-0.12; 0.06 | 36 | 7.50 | CI95%=-0.07; 0.11 | 0.93 | 0.56 | 1.56 |  |  |
| **rs3759467** | TT | 278 | 63.30 | p=0.005 | 312 | 65.40 | p=0.091 | 1^*^ |  |  | χ^2^_df=2_ =0.92  p=0.632 |  |
|  | TC | 154 | 35.10 | *f*=-0.133 | 155 | 32.50 | *f*=-0.085 | 1.11 | 0.85 | 1.47 |  |  |
|  | CC | 7 | 1.60 | CI95%=-0.20; -0.06 | 10 | 2.10 | CI95%=-0.16; -0.006 | 0.80 | 0.31 | 2.07 |  |  |
| ***BAFF-R***  ***TNFRSF13***  **polymorphisms** | | **Patients (N=439)** | |  | **Controls (N=477)** | |  | **OR** | **CI95%** | | **Patients vs. Controls** |  |
|  |  | **N** | **%** | **HWE** | **N** | **%** | **HWE** |  |  |  |  |  |
| **rs5996088** | GG | 361 | 82.20 | p=0.561 | 389 | 81.60 | p=0.300 | 1^*^ |  |  | χ^2^_df=2_ =2.41  p=0.308 |  |
|  | GA | 76 | 17.30 | *f*=-0.04 | 81 | 17.00 | *f*=0.05 | 1.01 | 0.72 | 1.43 |  |  |
|  | AA | 2 | 0.50 | CI95%=-0.10; 0.04 | 7 | 1.50 | CI95%=-0.05;0.15 | 0.36 | 0.09 | 1.51 |  |  |
| **rs7290134** | TT | 301 | 68.60 | p=0.254 | 340 | 71.30 | p=0.59 | 1^*^ |  |  | χ^2^_df=2_ =3.46  p=0.177 |  |
|  | TC | 121 | 27.60 | *f*=0.05 | 128 | 26.80 | *f*=-0.04 | 1.07 | 0.80 | 1.43 |  |  |
|  | CC | 17 | 3.90 | CI95%=-0.05;0.15 | 9 | 1.90 | CI95%=-0.11;0.05 | 2.08 | 0.93 | 4.65 |  |  |
| **rs6002551** ^a^ | CC | 379 | 86.3 | p=0.711 | 416 | 87.20 | p=0.46 | 1^*^ |  |  | χ^2^_df=2_ =1.15  p=0.64 |  |
|  | CT | 59 | 13.4 | *f*=-0.04 | 58 | 12.20 | *f*=0.03 | 1.12 | 0.76 | 1.64 |  |  |
|  | TT | 1 | 0.2 | CI95%=-0.08;0.043 | 3 | 0.6 | CI95%=-0.06;0.14 | 0.47 | 0.07 | 3.20 |  |  |

Abbreviations : OR, odds ratio; CI, confidence intervals; HWE, Test for Hardy-Weinberg equilibrium; *f*, departure from HWE * the reference group;

^a^ Wang et al. [7]

**A** WT His159


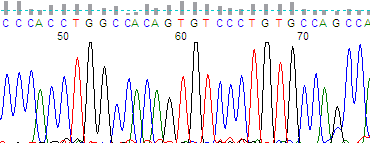


**B** His159Tyr


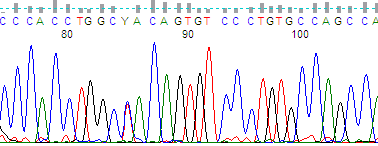


**C**

MW 1 2 3 4 5 6 7 8 9


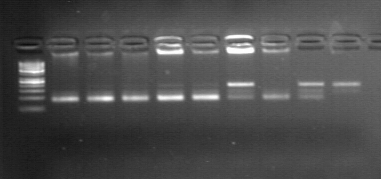


150 bp

172 bp

**Supplementary Figure 1.**

**Genotyping of rs61756766 C>T (His159Tyr) variant of *BAFF-R* gene.** (A) Sequence of wild type rs61756766 CC homozygote (His159). The black line indicates the TGG^CCA site recognized by *MlsI* (*MscI*) enzyme which was used for RFLP genotyping. (B) Sequence of rare variant of rs61756766 CT heterozygote (His159Tyr). The C to T (Y) substitution at rs61756766 introduces change in the TGGTCA site for *MlsI* (*MscI*) enzyme and causes that this site is not recognized by this enzyme. **(C)** Example of genotyping of rs61756766 C>T polymorphism by RFLP with the *MlsI (MscI*) restriction endonuclease*.* Lines 1,2,3,4,5 and 7 homozygotes CC; lines 6 and 8 heterozygotes CT; line 9 uncut PCR product of 172 bp. The *MlsI* (*MscI*) enzyme cuts the PCR product of CC homozygotes in TGG^CCA giving two products of 83 and 89 bp (viewed as one band on agarose gel). For CT heterozygotes only C allele is cut as the results two bands 172 bp and 83+89 can be seen on gel. MW (50bp Weight Marker).

Supplementary Table 4. Frequencies of *BAFF* and *BAFF-R* haplotypes among CLL patients and healthy controls.

| **Cases**  **[%]** | **Controls**  **[%]** | **rs9514827** | **rs3759467** | **rs1041569** | **rs9514828** |
| --- | --- | --- | --- | --- | --- |
| 34.7 | 34.6 | T | T | A | C |
| 26.0 | 25.3 | C | T | A | T |
| 17.5 | 18.0 | T | C | A | C |
| 13.7 | 15.8 | T | T | T | T |
| 3.0 | 3.0 | T | T | T | C |
| 2.2 | 1.6 | T | T | A | T |
|  |  |  |  |  |  |
| 97.1% | 98.3% | χ^2^_df=6_ = 4.4  p=0.622 | | | |
|  |  |  | | | |
| **Cases**  **[%]** | **Controls**  **[%]** | **rs5996088** | **rs61756766** | **rs7290134** | **rs6002551** |
| 81.4 | 82.7 | G | C | T | C |
| 6.6 | 5.6 | G | C | C | T |
| 6.3 | 7.6 | A | C | C | C |
| 2.4 | 1.0 | A | T | C | C |
| 2.3 | 0.9 | G | C | C | C |
| 0.4 | 1.1 | A | C | T | C |
|  |  |  |  |  |  |
| 99.4% | 98.9% | χ^2^_df=6_ = 17.4  p=0.008 | | | |
|  |  |  | | | |

Supplementary Table 5. Relationship between polymorphisms of *BAFF* gene and plasma BAFF concentration.

| **SNP** | **Genotype** | **N** | **Min** | **Q1** | **Median** | **S_n_** | **Q3** | **Max** |
| --- | --- | --- | --- | --- | --- | --- | --- | --- |
| **rs9514827** | TT | 87 | 56.16 | 230.3 | 338.9 | 143.3 | 452.4 | 3951 |
|  | TC | 75 | 112.1 | 205.7 | 316.1 | 160.9 | 466.6 | 2364 |
|  | CC | 5 | 220 | 298.4 | 367.8 | 111.9 | 443.9 | 516.9 |
| **rs3759467** | TT | 97 | 79.85 | 235.1 | 339.7 | 165.3 | 471.2 | 2364 |
|  | TC | 68 | 56.16 | 206.4 | 320.3 | 124.8 | 404.4 | 3951 |
|  | CC | 2 | 170.8 | 240.4 | 310 | 278.5 | 379.6 | 449.3 |
| **rs1041569** | AA | 109 | 96.46 | 219 | 335.9 | 146.1 | 470.6 | 2364 |
|  | AT | 50 | 56.16 | 171.1 | 316.5 | 165.5 | 444.1 | 3951 |
|  | TT | 8 | 186.2 | 288.1 | 343.6 | 65.1 | 364.4 | 463.7 |
| **rs9514828** | CC | 52 | 96.46 | 263.8 | 343.1 | 139.7 | 465.7 | 1334 |
|  | CT | 89 | 56.16 | 211.3 | 333.9 | 160.6 | 466.2 | 3951 |
|  | TT | 26 | 79.85 | 192.8 | 283.3 | 141.9 | 402.8 | 665.7 |
| F_8, 155_ = 1.154, p = 0.3308 | | | | | | | | |

**N=** number of patients**, Min –**Minimum, **Q1, Q3** – 1^st^ and 3^rd^ quartile; **S_n_** – variability measure; **Max**- Maximum

Supplementary Table 6. Relationship between polymorphisms of *BAFF* gene and intracellular expression of BAFF protein in peripheral blood CD19^+^ cells.

| **SNP** | **Genotype** | **N** | **Min** | **Q1** | **Median** | **S_n_** | **Q3** | **Max** |
| --- | --- | --- | --- | --- | --- | --- | --- | --- |
| **rs9514827** | TT | 68 | 0.32 | 4.722 | 11.53 | 9.93 | 25.12 | 93.29 |
|  | TC | 43 | 0.92 | 8.73 | 24.09 | 21.56 | 46.42 | 89.94 |
|  | CC | 10 | 0.95 | 9.432 | 14.14 | 12.62 | 27.72 | 86.34 |
| **rs3759467** | TT | 79 | 0.32 | 5.24 | 13.66 | 11.91 | 29.4 | 88.06 |
|  | TC | 39 | 0.79 | 8.755 | 17.81 | 16.23 | 36.45 | 93.29 |
|  | CC | 3 | 2.31 | 4.585 | 6.86 | 32.68 | 35 | 63.13 |
| **rs1041569** | AA | 83 | 0.79 | 6.205 | 13.95 | 13.08 | 30.64 | 89.94 |
|  | AT | 29 | 0.72 | 8.4 | 13.1 | 10.69 | 24.65 | 93.29 |
|  | TT | 9 | 0.32 | 3.07 | 11.59 | 13.23 | 32.54 | 83.6 |
| **rs9514828** | CC | 46 | 0.79 | 5.45 | 12.69 | 11.26 | 30.32 | 93.29 |
|  | CT | 53 | 0.72 | 6.51 | 14.12 | 14.01 | 30.59 | 89.94 |
|  | TT | 22 | 0.32 | 9.432 | 16.21 | 15.17 | 29.94 | 86.34 |
| F_8, 112_ = 1.166, p = 0.3257 | | | | | | | | |

**N=** number of patients**, Min –**Minimum **;Q1, Q3** – 1^st^ and 3^rd^ quartile; **S_n_** – variability measure, **Max**- Maximum

Supplementary Table 7. Association between the requirement for treatment and haplotypes of *BAFF* gene.

| **rs9514827** | **rs3759467** | **rs1041569** | **rs9514828** | **Frequency**  **[%]** | **OR** | **CI95%** | | **p-value** |
| --- | --- | --- | --- | --- | --- | --- | --- | --- |
| T | T | A | C | 34.58 | 1^*^ | - | - | - |
| C | T | A | T | 24.07 | 1.2 | 0.74 | 1.96 | 0.4648 |
| T | C | A | C | 16.74 | 1.55 | 0.88 | 2.73 | 0.1315 |
| T | T | T | T | 13.36 | 1.4 | 0.8 | 2.44 | 0.2454 |
| T | T | T | C | 4.05 | 1.53 | 0.62 | 3.79 | 0.3594 |
| T | T | A | T | 3.09 | 1.5 | 0.5 | 4.55 | 0.4735 |
| T | C | A | T | 1.64 | 0.95 | 0.18 | 5.18 | 0.9569 |
| C | T | A | C | 0.98 | 1.39 | 0.18 | 10.59 | 0.7509 |
| *others^**^* | | | | 1.49 | 0.39 | 0.04 | 3.58 | 0.4032 |
| χ^2^_df=8_ = 4.38, p = 0.8213 | | | | | | | | |

^*^ Baseline .

^* *^All haplotypes determined to be too rare to estimate their coefficients.

Supplementary Table 8. Association between time to treatment and haplotypes of *BAFF* gene.

| **rs9514827** | **rs3759467** | **rs1041569** | **rs9514828** | **Frequency**  **[%]** | **TTT**  **[months]** | **CI95%** | | **p-value** |
| --- | --- | --- | --- | --- | --- | --- | --- | --- |
| **T** | **T** | **A** | **C** | 35.03 | 5.82  mths^*^ | 2.75  mths | 12.34  mths | - |
| **C** | **T** | **A** | **T** | 20.02 | –14% | –59% | +79% | 0.6796 |
| **T** | **C** | **A** | **C** | 18.9 | –25% | –64% | +55% | 0.4362 |
| **T** | **T** | **T** | **T** | 14.57 | +20% | –44% | +155% | 0.6395 |
| **T** | **T** | **T** | **C** | 4.25 | –11% | –72% | +184% | 0.841 |
| **T** | **T** | **A** | **T** | 4.9 | –43% | –81% | +70% | 0.3203 |
| *others^**^* | | | | 2.34 | –88% | –98% | –31% | 0.0201 |
| χ^2^_df=6_ = 5.97, p = 0.4266 | | | | | | | | |

^**^ All haplotypes determined to be too rare to estimate their coefficients.

^*^ Baseline haplotype
